# A rheumatoid factor paradox: inhibition of rituximab effector function

**DOI:** 10.1186/ar4152

**Published:** 2013-01-25

**Authors:** Jonathan D Jones, Irene Shyu, Marianna M Newkirk, William FC Rigby

**Affiliations:** 1 Division of Rheumatology, Dartmouth Hitchcock Medical Center, One Medical Center Drive, Lebanon, NH 03756, USA; 2 Division of Rheumatology, McGill University Health Centre, 1650 Cedar Ave., Montreal, QC H3G 1A4, Canada

## Abstract

**Introduction:**

Rituximab (RTX) therapy of rheumatoid arthritis (RA) exhibits enhanced effectiveness in seropositive patients. Using patient sera, we tested if this improved efficacy was associated with enhanced RTX mediated complement-dependent cytotoxicity (RTX-CDC).

**Methods:**

We developed an *in vitro *assay for RTX-CDC using patient sera and the Daudi human B cell line. Using propidium iodide uptake and flow cytometry, we compared RTX-CDC with rheumatoid factor (RF)+ sera relative to normal volunteer, non-RA and RF- sera. Additional studies examined mixing studies of RF+ and RF- sera, as well as the effect of monoclonal IgA or IgM RF. Finally, the effect of RF on RTX mediated trogocytosis of normal B cells was evaluated.

**Results:**

Using human sera, addition of RTX resulted in rapid and profound (> 50%) Daudi cell death that was complement dependent. Surprisingly, RF+ patient sera exhibited reduced RTX-CDC relative to RF- sera, with an inverse relationship of RTX-CDC and RF titer. Mixing studies indicated the presence of an inhibitor of RTX-CDC in RF+ sera. The addition of monoclonal IgM or IgA RF to RF- sera markedly inhibited RTX-CDC. This effect was specific for RF binding to the Fc portion of RTX as it was not apparent with the F(ab)' domains of RTX engineered onto IgG3 heavy chain. RF also modestly inhibited RTX mediated trogocytosis.

**Conclusions:**

Contrary to expectations, RF+ sera exhibits reduced RTX-CDC due to the presence of RF. The enhanced efficacy of RTX in seropositive RA patients cannot be attributed to improved B cell depletion through CDC. This result indicates that high RF levels may potentially modulate the efficacy of any therapeutic monoclonal antibody dependent on Fc effector function.

## Introduction

Rheumatoid arthritis (RA) is a chronic, systemic inflammatory autoimmune disease that is classified as seropositive or seronegative based on the presence or absence of the autoantibodies rheumatoid factor (RF) and/or anti-citrullinated peptide antibodies (ACPA). Whereas RF is seen in various disease states, ACPA are relatively specific for RA [1]. These autoantibodies typically (approximately 95%) coexist and their presence predicts more erosive disease as well as increased cardiovascular morbidity in RA patients [2,3]. The nature of this high degree of coexistence of ACPA and RF remains uncertain. Does the high affinity RF characteristic of RA arise separately or is it due to the generation of immune complexes by ACPA? Further questions arise from the finding that the presence of ACPA and RF predict clinical responses to rituximab therapy in RA [4-6]. The pathophysiology of each of these associations remains unclear.

RF activity defines an antibody capable of binding the Fc portion of IgG in the γ2-γ3 cleft [7,8]. Interestingly, the heavy and light chain genes encoding low affinity RF antibodies arise from unmutated germ line immunoglobulin genes [9]. As a result, B cells capable of producing low affinity IgM RF are detected at a high frequency in the circulation of healthy volunteers [10,11]. The high levels of IgM RF seen in cryoglobulinemia typically arise from these germ line immunoglobulin genes [12]. Given their presence in the germ line and their specificity for the Fc portion of IgG, it has been generally assumed that low affinity IgM RFs have been evolutionarily selected for roles in binding and clearing immune complexes (circulating RFs) and/or endocytosing IgG containing complexes by RF+ B-cells so as to stimulate an anti-pathogen response [13,14].

In contrast to RFs seen in healthy controls and patients with cryoglobulinemia, the RFs associated with RA are high affinity and frequently have undergone class switching from IgM to IgA and IgG (reviewed in [15,16]). For these reasons, it is inferred, but unproven that these RF-producing plasma cells derived from B cells that have undergone T cell-dependent affinity maturation. In fact, it was the high affinity nature of RA-associated RF that permitted their initial characterization due to their ability to form immune complexes in blood *in vivo *[17]. Subsequent studies have demonstrated that these RF-containing immune complexes are also found in the synovial fluid and cartilage of RA patients [18,19]. Moreover, local RF production has been demonstrated in RA synovium, so it is likely that tissue levels of RF are higher than that seen in the circulation [20,21].

Despite these insights, the pathophysiologic role of RF remains unclear. In a classic *in vivo *study, administration of plasma containing high-titer RFs to humans did not result in articular signs or symptoms [22]. Early laboratory studies identified that RF was capable of complement activation via the classical pathway, suggesting that RF-immune complexes promote inflammation through complement activation [23]. Consistent with this concept, levels of RF immune complexes and complement in synovial fluid are inversely related [18,24]. However, in a separate study, RF was shown to be very poor at fixing C4 and C3 [25]. Thus, the precise role of RF in promoting or sustaining complement-induced inflammation is unclear. Understanding these issues is central to understanding the role of RF in RA.

These unresolved issues have taken on greater importance with the advent of antibody-based therapeutics, most notably with agents like the anti-CD20 monoclonal antibody rituximab, where complement-dependent cytotoxicity (CDC) might be important for the elimination of pathogenic B cells (reviewed in [26]). As previously stated, the presence of seropositivity predicted improved responses to rituximab (RTX) [4-6]. Thus, we hypothesized that these observations might be linked through the ability of RF to potentiate B cell depletion by RTX by enhancing its ability to mediate CDC (RTX-CDC).

We addressed this question using human sera from RA patients and various controls in an *in vitro *model of RTX-CDC. Unexpectedly, a series of findings led us to conclude that RF inhibits, rather than potentiates, RTX-CDC. RF does not block RTX binding to the B cell, suggesting that RF blocks the effector function of its Fc portion. Consistent with this interpretation, we also show that RF inhibits RTX-mediated trogocytosis, an FcγR-dependent effect [27,28]. These surprising results relate not only to the consideration of rituximab efficacy in RA, but also illustrate a more complex interaction of RF, IgG, and complement than previously appreciated.

## Materials and methods

### Reagents

Serum-free media X-Vivo 15 and Aim-V were purchased from Lonza (Walkersville, MD, USA) and Gibco (Life Technologies, Grand Island, NY, USA), respectively. Purified human IgG and propidium iodide (PI) were purchased from Sigma-Aldrich (St. Louis, MO, USA). Mouse anti-human CD45 FITC and mouse anti-human CD20 APC-Cy7 were from BD Biosciences (San Jose, CA, USA) and mouse anti-human CD19 APC was from BioLegend (San Diego, CA, USA). Rabbit polyclonal anti-human C1q FITC was purchased from Abcam (Cambridge, MA, USA). Rituximab (Genentech, San Francisco, CA, USA)), a mouse:human chimeric antibody with the human IgG1 heavy chain, and eculizumab (Alexion, Cheshire, CT, USA), a monoclonal antibody binding complement component C5, were obtained from the hospital pharmacy. A genetically modified form of rituximab engineered to contain the human IgG3 heavy chain (referred to in the text as IgG3 RTX) was purchased from InvivoGen (San Diego, CA, USA). Monoclonal IgM RF was purified from the ascites from a patient with mixed cryoglobulinemia as previously reported [29]. Monoclonal IgA RF was a synthetic molecule with the V-region genes based on the sequence of RF61 [30] fused to the IgA constant region, kindly provided by Cayla InvivoGen (Toulouse, France). Mouse anti-human C3b/iC3b (2H11) was a kind gift from Ron Taylor (University of Virginia) and was labeled with Alexa-fluor 488 according to the manufacturer's instructions (Life Technologies, Grand Island, NY, USA).

### Cells/sera

The Daudi human B cell line was adapted to and maintained in the serum-free media X-Vivo 15 in a 5% CO_2 _incubator at 37°C. Healthy donor and patient sera were obtained following written consent to a protocol approved by the Committee for the Protection of Human Subjects at Dartmouth College. Blood was drawn into sterile glass tubes, allowed to clot and the clot pelleted by 15-minute centrifugation (700 g). Sera was aspirated, aliquotted and frozen at -80°C until shortly before use. Sera were obtained from healthy donors (*n *= 15), non-RA patients (*n *= 15), seronegative RA patients (*n *= 15), and seropositive RA patients (*n *= 40). All seropositive and seronegative RA patients had established disease for more than two years. Non-RA patients included four patients with osteoarthritis, psoriatic arthritis, with vasculitis/polymyalgia rheumatica (PMR), two with an undifferentiated seronegative inflammatory arthritis, and one with arthropathy associated with celiac disease.

Blood was obtained from healthy volunteer donors following informed consent and peripheral blood mononuclear cells (PBMC) purified by discontinuous gradient isolation using Ficoll-Paque PLUS (GE Healthcare Biosciences, Uppsala, Sweden). B cells were isolated by negative selection, using Invitrogen's Untouched B-Cell Isolation Kit (#113.51d, Life Technologies, Grand Island, NY, USA). CD20 density on cell surfaces was determined by pelleting healthy PBMC and Daudi cells, staining with anti-CD20 APC-Cy7 on ice for 30 minutes, fixation with 1% formaldehyde, followed by CD20 mean fluorescent intensity (MFI) measurement using MACSQuant Analyzer (Miltenyi Biotec, Boston, MA, USA), and interpretation using FlowJo software (TreeStar, Ashland, OR, USA).

### Rheumatoid factor status

Seropositivity was determined by chart review for individual patient sera and typically reflected that obtained by nephelometry. Seropositivity for IgM RF and IgA RF was confirmed and quantified using an ELISA (TheraTest, Lombard, IL, USA). Three donors who were originally classified as seronegative RA by chart review yielded low levels of RTX-CDC and were subsequently confirmed to have IgA and IgM RF by ELISA, and were re-classified as seropositive for the purposes of this study.

### Rituximab complement-dependent cytotoxicity (RTX-CDC) assay

RTX (0.1 to 10 μg/ml) was added to Daudi cells in X-Vivo 15, followed by immediate addition of human serum (1, 2 or 5%) in round bottom flow cytometry tubes (BD Falcon, BD Biosciences, San Jose, CA, USA), at a final concentration of 250,000 cells/ml, incubated for 30 minutes at either RT or 37°C followed by addition of propidium iodide (PI-1 μg/ml final concentration). Flow cytometry was utilized to determine dead cells by PI uptake (FACScan, BD Biosciences, San Jose, CA, USA) after a 30-minute incubation. Complement-independent effects on Daudi cell viability were assayed using RTX in serum-free X-Vivo 15 alone, with 5% C5 deficient serum (Sigma-Aldrich, St. Louis, MO, USA), or 5% heat-inactivated serum (56°C for 45 minutes), compared to 5% normal human serum and assayed as described above.

### Inhibition of RTX-CDC by rheumatoid factor

Mixing studies were performed using seronegative serum with RF positive serum, each at 1% of total volume, prior to addition of RTX. Similarly, monoclonal IgM or IgA RF was added to 1% serum prior to addition of RTX and then Daudi cells. Assessment of Daudi cell viability was performed as above.

### RTX blocks C3b and C1q deposition on B cells

Highly enriched human B cells (approximately 97% purified) obtained from healthy donors were cultured at a concentration of 1 × 10^6 ^cells/ml in RPMI + 10% normal human serum with RTX at 0.1, 1, and 10 μg/ml in the presence or absence of 10 μg/ml monoclonal IgM RF at RT for 30 minutes, followed by a wash with ice cold PBS/BSA/Azide. After pelleting, the cells were stained with Al-488 labeled anti-C3b/iC3b antibody for 30 minutes on ice, washed and then fixed with 1% paraformaldehyde. C3b deposition on cells was determined by flow cytometry using FACSCalibur (BD Biosciences, San Jose, CA, USA).

Daudi cells were subjected to the standard RTX-CDC assay as outlined above, using either healthy donor serum or RF+ serum. Prior to addition of RTX, half of the cells were incubated with eculizumab 10 μg/ml, a C5 inhibitor, for 15 minutes, to prevent CDC but to still allow for C1q fixation. After a 30-minute incubation at RT, the cells were washed with ice cold PBS/BSA/Azide, pelleted and stained with FITC labeled rabbit polyclonal anti-human C1q antibody for 30 minutes on ice. After another wash, the cells were fixed with 1% paraformaldehyde and C1q deposition was determined by flow cytometry using FACSCalibur.

### RF binds to RTX after cell attachment to inhibit CDC

To discriminate the mechanism by which RF blocked RTX-CDC, the effect of preincubating (15 minutes) Daudi cells with RTX (10 μg/ml) to bind cell surface CD20 prior to the addition of 1% serum and IgM RF 50 μg/ml was compared with combining RTX, IgM RF and serum prior to adding Daudi cells. Cell viability was measured as described above. Similarly, the effect of RF on RTX engagement of CD20 was measured by incubating Daudi cells with RTX 10 μg/ml alone, IgM RF 50 μg/ml alone, or a combination of RTX and IgM RF for 30 minutes followed by staining with mouse anti-human CD20 APC-Cy7 and evaluated by flow cytometry (FACSCanto, BD Biosciences, San Jose, CA, USA).

### IgM RF blocks IgG1 effector function, but not IgG3

As a comparison to standard RTX (IgG1), IgG3 RTX (InvivoGen) was used in the RTX-CDC assay. RF positive sera were used in parallel with RF negative sera at 2% of total volume. Alternatively, 2% RF- sera was utilized in the RTX-CDC assay with or without the addition of 50 μg/ml monoclonal IgM RF. Due to limited quantities of IgG3 RTX, a concentration of 1 μg/ml was utilized instead of 10 μg/ml.

### Effect of RF on RTX trogocytosis

Blood, obtained from healthy volunteer donors following informed consent, and PBMC, purified by discontinuous gradient isolation using Ficoll-Paque PLUS (GE Healthcare Biosciences), were resuspended in Aim V serum-free media (Gibco) at 2 million cells/ml. PBMC were then combined with no RF, 10 μg/ml IgM or IgA RF, or 100 μg/ml IgM or IgA RF. The PBMC were either untreated, or were treated with RTX 10 μg/ml for 30 minutes at RT. The cells were then washed in cold PBS/BSA/Azide, pelleted, then stained with anti-CD19 APC and anti-CD45 FITC for 30 minutes on ice. After another wash, the cells were pelleted and fixed with 1% paraformaldehyde, followed by flow cytometry using FACSCalibur.

### Statistics

Statistical analysis was performed using STATA software (StataCorp, College Station, TX, USA). Error bars represent standard error of the mean. Statistical significance of IgM and IgA RF concentration to degree of CDC was determined using linear regression. Where appropriate, comparison of two means was performed using the *t*-test, with a *P *< 0.05 considered statistically significant.

## Results

### Development of a RTX-CDC assay

**Figure 1:**
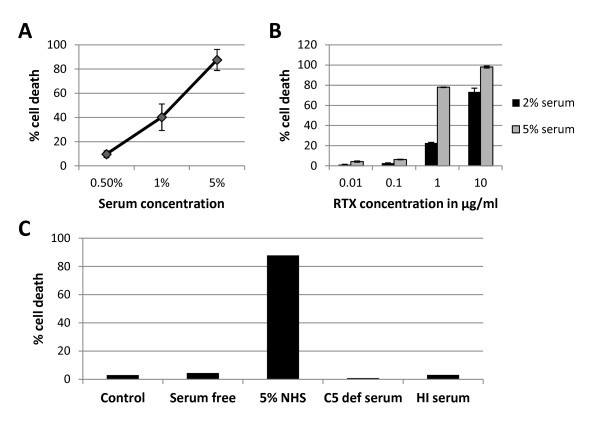
**RTX complement-dependent cytotoxicity (CDC) induced death of Daudi human cell line**. **A**. Effect of varying concentration of normal human serum (NHS) with RTX 10 μg/ml (*n *= 3) as determined by propidium iodide staining. **B**. Effect of varying RTX concentration with 2% and 5% NHS (*n *= 3). Error bars represent mean standard error. **C**. Daudi cell death with 5% NHS represents CDC as evidenced by lack of cell death in complement free conditions (serum free media, C5 deficient serum, and heat inactivated (HI) serum).

RTX mediates B cell depletion through various mechanisms, including CDC [26]. To test the hypothesis that RF might enhance RTX-CDC, we developed an *in vitro *assay with the human Daudi B cell line and human sera. Since determining that cell viability is the best measure of CDC [31], we used propidium iodide (PI) uptake to determine the amount of RTX-CDC present. There were no differences in RTX-CDC assay performed at RT and at 37°C, so subsequent experiments were performed at RT (*n *= 6, data not shown). Under these conditions, RTX (10 μg/ml) mediated rapid (minutes) and significant cell death (40%) with only 1% normal human serum (*n *= 3, Figure 1A). Nearly complete cell death (88%) was observed with 5% serum. Dose titrations of RTX in the presence of 2% and 5% serum showed cell death of 23% and 78%, respectively, at 1 μg/ml (*n *= 3, Figure 1B). This difference between 2% and 5% narrowed to 73% and 98% cell death using RTX at 10 μg/ml. All observed Daudi cell death was complement-dependent; there was none seen using RTX (10 μg/ml) with serum free media, heat-inactivated serum (56°C, 45 minutes), or C5 deficient serum (Figure 1C).

### Disease-associated variations in RTX-CDC

**Figure 2:**
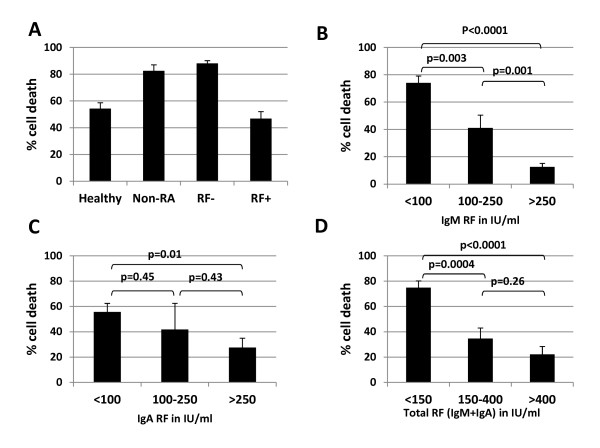
**RTX-CDC varies as a function of patient sera and correlates with RF levels**. **A**. RTX-CDC using Daudi cell line with 1% human sera stratified by disease state: healthy donors, non-RA disease, seronegative RA (RF-), and seropositive RA (RF+). Data represent the mean cell death obtained with 15 sera in each category except for RF+ (*n *= 40). **B, C, D**. RTX-CDC cell death declines with increasing titer of RF, with a stronger correlation with IgM RF. Error bars represent mean standard error.

**Figure 3:**
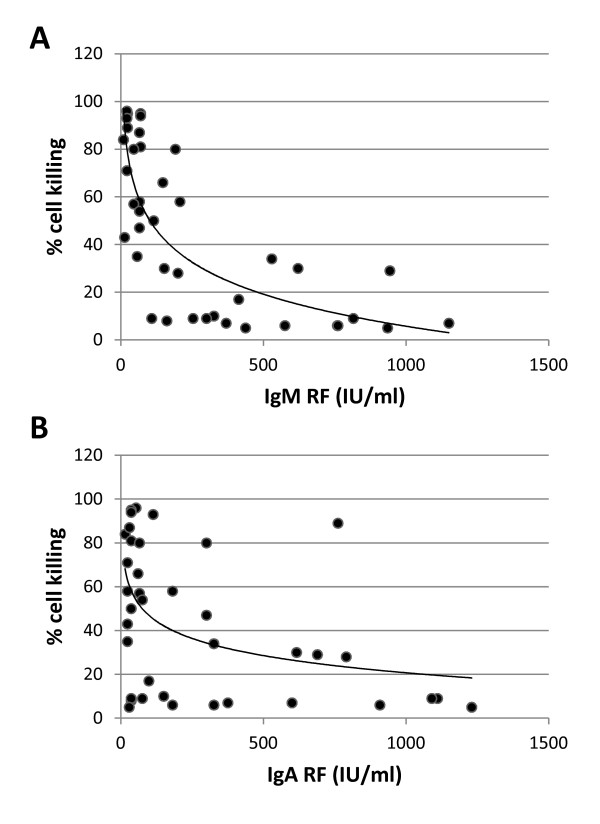
**Dot-plots of RTX-CDC of Daudi cells as a function of RF concentration (IU/ml)**. Using RTX 10 μg/ml and 1% serum, CDC was determined as a function of **A**. IgM RF concentration (*n *= 40, R^2 ^= 0.45, *P *< 0.0001) and **B**. IgA RF concentration (*n *= 40, R^2 ^= 0.18, *P *= 0.006).

We hypothesized that the presence of RF in human sera would enhance RTX-CDC. To investigate this, we measured Daudi cell death using sera from four different populations: healthy donors (*n *= 15), non-RA patients (*n *= 15), seronegative RA patients (*n *= 15), and seropositive RA patients (*n *= 40). Using limiting serum concentrations (1%), the average cell death in sera from healthy donors and seropositive RA donors was very similar (54% and 47%, respectively, *P *= 0.28) (Figure 2A), though greater variability was seen using sera from seropositive patients. Interestingly, the RTX-CDC seen with non-RA patients and seronegative RA patients was significantly higher (*P *< 0.0001). While it was not surprising that sera from patients with systemic inflammation had higher RTX-CDC than healthy donors, the reduction in RTX-CDC and the wide variability of RTX-CDC seen with sera from seropositive RA patients was completely unexpected. This apparent incongruity became clear upon finding that stratification of RTX-CDC as a function of RF titer clearly showed an inverse correlation (Figure 2B-D). The correlation held true with IgA RF (R^2 ^= 0.18; *P *< 0.006), but was stronger for IgM RF (R^2 ^= 0.45; *P *< 0.0001) (Figure 3).

### Seropositive sera inhibits CDC when mixed with seronegative sera

**Figure 4:**
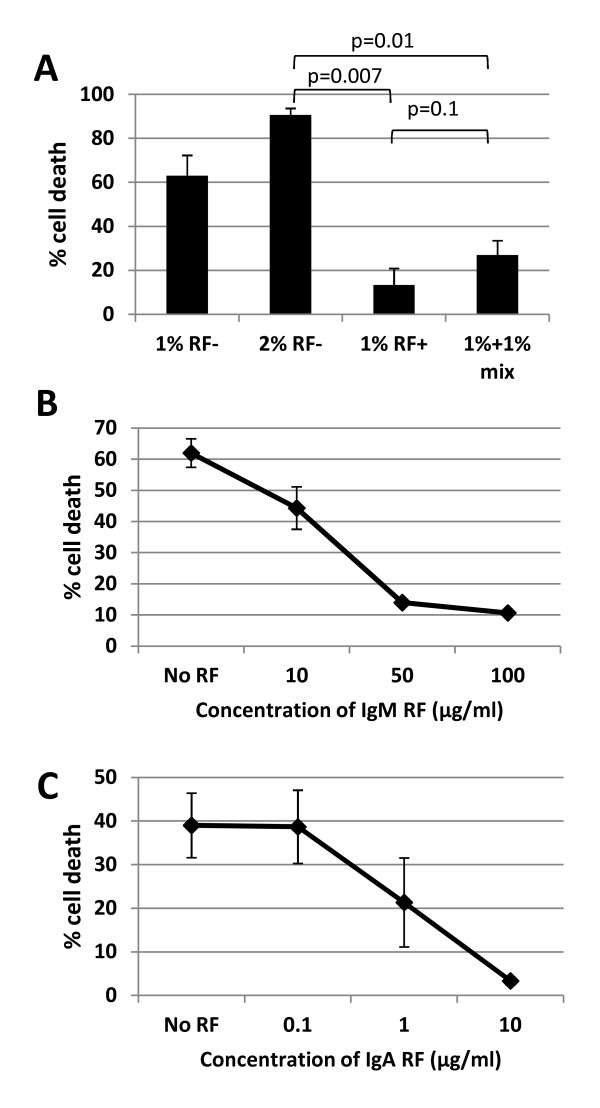
**Inhibition of RTX-CDC is mediated by RF**. **A**. Mixing studies using seronegative (RF-) and seropositive (RF+) sera demonstrates inhibition of CDC by RF+ sera, indicating that an inhibitor, not a factor deficiency, decreases CDC. **B**. Addition of monoclonal IgM RF markedly inhibits RTX-CDC by NHS. **C**. Addition of monoclonal IgA RF markedly inhibits RTX-CDC by NHS. Graphs represent average of three experiments; error bars represent mean standard error.

One possible mechanism for this observation was the possible association of RF titer with reductions in serum complement. If true, a mixing study with normal human serum would be expected to correct the reduction in RTX-CDC. The mixing of 1% serum from a seropositive patient with those from seronegative patients showed the opposite result: RF+ sera inhibited RTX-CDC (*n *= 3, Figure 4A). Thus, the decreased RTX-CDC associated with RF+ sera operated in *trans *to block RTX-CDC.

### Rheumatoid factor inhibits RTX-CDC

These data indicated the presence of an inhibitor in RF+ sera. The potential role of RF itself binding to the Fc portion of rituximab was supported by finding that a monoclonal IgM RF markedly reduced RTX-CDC (*n *= 3), becoming maximal at 50 μg/ml (Figure 4B). A similar effect was seen with a monoclonal IgA RF (Figure 4C, *n *= 3).

### Rheumatoid factor blocks C3b deposition on normal B cells and C1q deposition on Daudi cells

**Figure 5:**
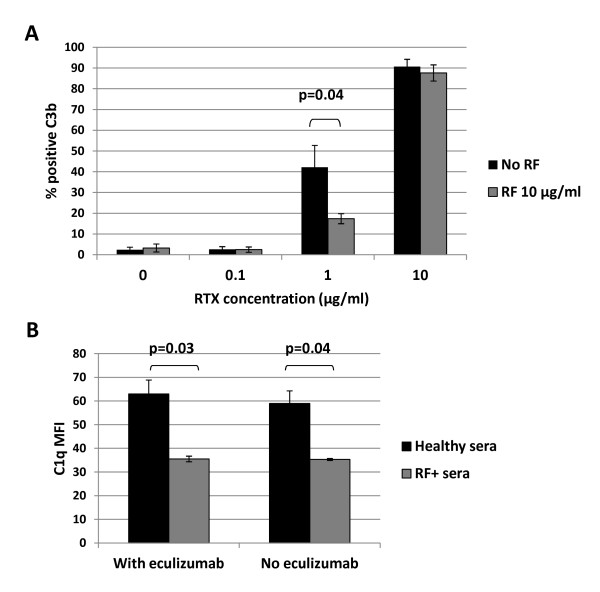
**Rheumatoid factor blocks C1q and C3b deposition**. **A**. Enriched normal human B cells were treated with RTX at varying concentrations in the presence or absence of IgM RF 10 μg/ml for 30 minutes, and then stained for C3b (*n *= 5). **B**. Daudi cells were treated with RTX 10 μg/ml for 30 minutes in the presence of healthy serum or RF+ serum, with or without eculizumab 10 μg/ml, followed by staining for C1q (*n *= 3). Mean fluorescence intensity (MFI) was determined by flow cytometry. Error bars represent mean standard error.

Normal human B cells were used as an alternative model to study the role of RF on rituximab function. We have previously shown that, in contrast to the Daudi lymphoblastoid cell line, human peripheral blood cells B cells are resistant to RTX-CDC [27]. The nature of this resistance is unclear; Daudi cells express higher levels (20×) of surface CD20 (data not shown), but are also much larger. Thus, instead of measuring cell death to assess modulation of rituximab activity by RF, we measured deposition of C3b on the B cell surface. Using highly enriched B cells (approximately 97% purified), RTX was added in the presence or absence of monoclonal IgM RF, followed by measurement of C3b deposition by flow cytometry (*n *= 5). These studies revealed that RF inhibited RTX dependent C3b deposition (Figure 5A).

To confirm the hypothesis that RF blocks CDC by limiting C1q binding to RTX-CD20 complexes, Daudi cells were combined with RTX and sera from healthy donors or RF+ donors, followed by measurement of C1q deposition by flow cytometry. Figure 5B illustrates that RF+ sera resulted in a nearly 50% inhibition of C1q binding (*n *= 3, *P *< 0.05), which was unaffected by eculizumab.

### Rheumatoid factor blocks complement fixation of RTX after RTX engagement of CD20 on the cell surface

**Figure 6:**
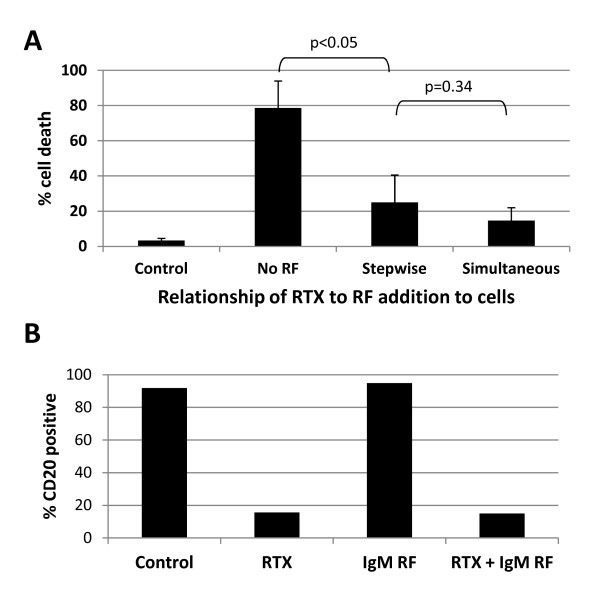
**IgM rheumatoid factor does not prevent RTX attachment to CD20**. **A**. RF inhibits B cell death whether RTX is first added to cells followed later by RF addition (Stepwise), or if RTX and RF are added simultaneously (*n *= 3). Error bars represent mean standard error. **B**. Representative data (*n *= 2) showing that RTX blocks binding of anti-human CD20 APC-Cy7, and addition of RF to RTX does not lessen this blocking outcome.

The effect of RF on RTX-CDC (and C1q deposition) might be due to its capture of RTX in fluid phase, thus preventing binding to CD20 on the B cell. Alternatively, RF engagement of the Fc domain of RTX might block the recruitment of complement after RTX had engaged with CD20. To investigate these possibilities, we compared the resultant CDC from preincubation (15 minutes) of Daudi cells with RTX (to ensure B cell binding) followed by addition of IgM RF and serum relative to the simultaneous addition of RTX, RF and serum (Figure 6A). RF blocked RTX-CDC independent of the time of addition (stepwise vs. simultaneous) (*n *= 3, *P *= 0.34). These data suggest that RF inhibits complement binding to the Fc portion of RTX after it has bound CD20. This interpretation was supported by the observation that RF had no effect on RTX binding to CD20, as measured by the lack of modulation of the ability of RTX to block the binding of fluorochrome-labeled anti-human CD20 (APC-Cy7) to Daudi cells (Figure 6B). Thus, RF does not limit the ability of RTX to bind CD20 on the B cell. These data suggest that RF inhibits C3b deposition and cytotoxicity by preventing C1q binding to the Fc portion of RTX.

### Rheumatoid factor inhibits IgG effector function through binding the Fc portion of RTX

**Figure 7:**
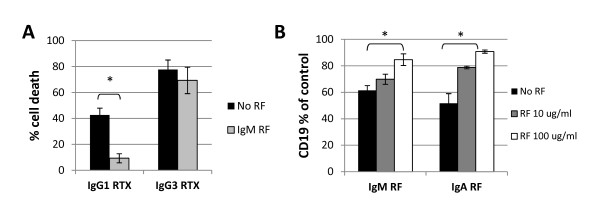
**RF inhibits rituximab effects by binding the Fc portion of RTX**. **A**. Monoclonal IgM RF has an inhibitory effect on RTX-CDC when RTX (1 μg/ml) has an IgG1 heavy chain, but not when RTX has an IgG3 heavy chain (*n *= 3). **B**. Addition of RTX 10 μg/ml to PBMC in serum free media after addition of monoclonal IgM RF (*n *= 6) or IgA RF (*n *= 3) inhibits loss of CD19 by RTX-mediated trogocytosis. Inhibitory effects of RF found to be statistically significant (*P *< 0.05) are indicated by asterisks. Error bars represent mean standard error.

IgM RF reacts with the cleft in the Cγ2/Cγ3 Fc portion of human IgG1, IgG2 and IgG4, but not IgG3 [7,8]. To prove the observed inhibitory effect of IgM RF requires a specific Fc domain, we compared RTX-CDC relative to IgG3 RTX. IgG3 RTX differs from RTX only in the presence of an IgG3 instead of IgG1 heavy chain. IgG3 RTX mediated RTX-CDC more efficiently than IgG1 RTX. More importantly, the addition of monoclonal IgM RF to RF- sera (*n *= 3) inhibited CDC with IgG1 RTX, but not with IgG3 RTX (Figure 7A).

To further confirm that RF modulates the ability of the Fc portion of RTX to mediate effector function, we evaluated an alternate activity (trogocytosis) of the Fc portion of RTX using normal human B cells. Rituximab binds the surface of normal human B cells and mediates loss of CD19 and CD20 through engagement of the Fc portion of RTX by effector cells expressing FcγR [27,28]. This process is complement-independent and not associated with cell death [27]. We examined trogocytosis with RTX using PBMC from healthy donors, measuring the degree of CD19 loss by flow cytometry in the absence or presence of RF. Both IgM (*n *= 6) and IgA RF (*n *= 3) inhibited CD19 loss by trogocytosis to a moderate degree (Figure 7B).

## Discussion

We report the unexpected result that RF inhibits the ability of RTX to mediate CDC of human B cells. This effect is not associated with decreased RTX binding to CD20 on B cells, suggesting that RF sterically inhibits the ability of the Fc domain of RTX to bind and/or recruit the proximal components of the complement cascade, namely C1q [32]. To a lesser degree, RF additionally blocks the ability of RTX to mediate 'shaving' or trogocytosis of CD19, an Fc-dependent process [27,28]. The requirement for RF binding to the Cγ2/Cγ3 cleft in IgG1, IgG2 and IgG4 for this inhibition was confirmed by the lack of effect of RF on CDC by a variant of RTX containing the IgG3 heavy chain [7].

This effect on RTX-CDC was seen with both monoclonal IgM and IgA RF and exceeded their effect on trogocytosis. This observation corresponds to the relative distance of these effector domains (C1q binds Cγ2; FcγR binds the upper Cγ2 + hinge regions) from the RF binding site in the Cγ2/Cγ3 cleft [32-35]. Thus, steric hindrance by RF of C1q binding is greater than FcγR. The observation that IgA RF appeared more effective than IgM RF at decreasing RTX-CDC, but not trogocytosis, is potentially interesting and suggests additional interactions that require further study.

Clinical trials indicate that rituximab exhibits superior efficacy in the treatment of seropositive RA relative to its seronegative counterpart, particularly in the presence of IgA RF [4]. Based on animal models of rituximab action, we postulated that RTX-CDC would be required for the depletion of non-circulating pathogenic synovial B cells [36]. In this model, we expected that local production of RF in synovium might potentiate RTX-CDC. However, our results show the opposite effect, RF inhibits RTX-CDC. These results enhance our understanding of the mechanism of action of RTX *in vivo*, in that they suggest that RTX-CDC does not account for the increased efficacy of RTX in seropositive patients. The potential impact of C3a and C5a generation through complement fixation by RTX was not specifically measured.

In contrast, we also demonstrated that RF decreased the ability of phagocytic cells to mediate 'shaving' or trogocytosis of CD19 from normal human B cells, which is an FcγR-dependent process. Thus, the presence of RF might prevent the loss of the CD20-RTX complex by trogocytosis, enabling enhanced levels of B cell depletion by antibody dependent cellular cytotoxicity (ADCC) to occur. However, this activity of RF would also be predicted to block ADCC, although it is possible that ADCC and trogocytosis might be differentially modulated by RF binding. Finally, our studies may underestimate the magnitude of the effect of RF in an inflamed joint given its high concentrations that arise from local production [20,21].

These findings suggest a need for a more general re-examination of RF biology, as the literature is unclear on this topic. Animal models have shown that RF promotes immune complex clearance presumably through the formation of larger complexes and fixation of proximal components of the complement cascade [37,38]. Moreover, clinical samples have shown lower levels of complement (C3 and CH_50_) in association with higher levels of RF and immune complexes [18]. However, direct measures indicate that IgM RF is poor at fixing complement [25]. Our findings are consistent with this latter finding and prompt us to ask: If RF blocks effector function in terms of C1q and FcγR binding, how are these activities reconciled with its ability to enhance immune complex clearance?

## Conclusions

We report the surprising finding that RF inhibits several RTX effector functions (complement dependent cytoxicity, FcR-mediated trogocytosis of CD19). These effects are attributable to the ability of RF to bind to the Cγ2/Cγ3 cleft of the Fc portion of rituximab and sterically inhibit both C1q binding and FcγR engagement. These data suggest a surprising paradox as they are the opposite results that one would predict given the superior efficacy in seropositive RA. However, the implications of this binding potentially extend beyond rituximab to any antibody-based therapeutic in RA, particularly those in which Fc-dependent function is important for clinical benefit.

## Abbreviations

ACPA: anti-citrullinated protein antibodies; ADCC: antibody dependent cellular cytotoxicity; APC: allophycocyanin; BSA: bovine serum albumin; CDC: complement dependent cytotoxicity; ELISA: enzyme linked immunosorbent assay; FITC: fluorescein isothiocyanate; MFI: mean fluorescent intensity; NHS: normal human serum; PBMC: peripheral blood mononuclear cells; PBS: phosphate buffered saline; PI: propidium iodide; PMR: polymyalgia rheumatica; RA: Rheumatoid arthritis; RF: Rheumatoid factor; RT: room temperature; RTX: rituximab; RTX-CDC: rituximab mediated complement dependent cytotoxicity

## Competing interests

The authors declare that they have no competing interests.

## Authors' contributions

JDJ had a primary role in concept development, data acquisition and evaluation, and in drafting and revising the manuscript. IS contributed to collection of patient sera and data evaluation. MMN contributed to data acquisition and evaluation, and revision of the manuscript. WFCR had a primary role in concept development, data evaluation, and in revision of the manuscript. All authors read and approved the final manuscript.
